# Socio-economic inequities in mental health problems and wellbeing among women working in the apparel and floriculture sectors: testing the mediating role of psychological capital, social support and tangible assets

**DOI:** 10.1186/s12889-024-18678-5

**Published:** 2024-04-25

**Authors:** Karen Schelleman-Offermans, Bilisuma B. Dito, Konjit H. Gudeta, Elsje Fourie, Sindu W. Kebede, Valentina Mazzucato, Kai J. Jonas

**Affiliations:** 1 Department of Work and Social Psychology, Faculty of Psychology and Neuroscience, Maastricht University, Postbus 616, Maastricht, 6200MD the Netherlands; 2 Department of Society Studies, Faculty of Arts and Social Sciences, Maastricht University, Maastricht, the Netherlands; 3 School of Commerce, Addis Ababa University, Addis Ababa, Ethiopia; 4 FRONTIERi, Berlin, Germany

**Keywords:** Health inequalities, Socio-economic position, Mental health, Wellbeing, Psychological capital, Social support, Reserve capacity model, Foreign direct investment, Female workers

## Abstract

**Background:**

It is still unknown whether the mechanisms proposed by the Reserve Capacity Model (RCM) explaining socio-economic health and wellbeing inequities in high income countries can be applied to low-income countries. This study investigates whether different reserve capacities (intra-, inter-personal, and tangible) can explain the association between relative socio-economic position (SEP) and wellbeing outcome measures among Ethiopian women working in Foreign Direct Investment (FDI).

**Method:**

Using a cross-sectional design, we collected quantitative survey data among 2,515 women working in the apparel and floriculture sectors in Ethiopia, measuring GHQ-12 mental health problems, multi-dimensional wellbeing, relative SEP, psychological capital (PsyCap), social support (emotional and financial social support network), and tangible assets (e.g., owning mobile phone, having access to toilet facilities). We used cluster-adjusted structural equation modelling to test whether PsyCap, social support, and/or tangible assets mediate the association between relative SEP (IV) and GHQ-12 mental health problems and multi-dimensional wellbeing (DVs).

**Results:**

PsyCap and the size of the financial support network significantly mediate the socio-economic gradient in both wellbeing outcomes. The size of the emotional social support network shows no association with multi-dimensional wellbeing and shows an unexpected negative association with GHQ-12 mental health problems scores, including a significant mediation effect. Tangible assets show no association with the wellbeing outcome measures and do not mediate socio-economic mental health problems and wellbeing inequities.

**Conclusions:**

The RCM can be applied in low-income countries, although in unexpected ways. Similar to findings from high-income countries, PsyCap and size of the financial social support network show significant mediation effects in explaining mental health problems and wellbeing inequities in Ethiopia. These reserves could therefore serve as a buffer for socio-economic inequities in mental health and wellbeing and can therefore assist in decreasing these inequities for women working in FDI sectors in Ethiopia.

**Supplementary Information:**

The online version contains supplementary material available at 10.1186/s12889-024-18678-5.

## Introduction

Reducing socio-economic inequities in health and wellbeing is one of the biggest challenges that the field of public health [1] faces in its contribution to sustainable development. For this reason, the goals of ‘reducing inequities within and among countries’ (Sustainable Development Goal (SDG) 10) and ‘ensuring healthy lives and promoting wellbeing for all and at all ages’ (SDG3) have been included in the 2030 Agenda for Sustainable Development by the World Health Organization [2]. Although the problem of health inequities has received considerable attention since the 1980s [3], large differences in the prevalence and incidence of somatic health disorders (e.g., stroke, diabetes, lung cancer) as well as mental disorders (e.g., depression) still persist between people with a low and high socio-economic position (SEP) globally [4–6]. Investigating the determinants of inequities in health and wellbeing is therefore crucial, especially among vulnerable populations [7]. However, research on the applicability of theoretical models on the etiological pathways leading to health inequities, stem mainly from Western, industrialized high-income societies such as the United Kingdom, United States or European countries, in which individual needs are more stressed than group needs [5, 8–12]. It is still unknown whether the same explanatory mechanisms apply in countries in the Global South, in which group needs are often more emphasized compared to individual needs and where the overall GDP is lower. Furthermore, to decrease health inequities within a country, it is crucial to specifically target the vulnerable populations within the country [7]. Gender inequality in health is especially high among women in the Global South [13], increasing the vulnerable position for females in this context. Therefore, it is important to investigate these explanatory mechanisms specifically for women working in low-waged jobs within the context of Ethiopia. The main aim of the current study is therefore to gain more insight into the applicability of existing theories tested in Western high-income countries to women working in FDI in Ethiopia.

### The context of Ethiopia

Ethiopia is the largest country in the Horn of Africa [14]. Although exports and FDI have stagnated since 2020–2021, mainly due to the Covid-19 pandemic and an ongoing civil war in large parts of the country, the country showed one of the steepest increases in economic growth in the Global South over the preceding decade [14, 15]. Government support, low wages, and a large potential workforce contributed to an increase in FDI, especially in the apparel and floriculture sectors. This created a large increase in new jobs, the majority of which were filled by women [15]. For many of the Ethiopian women working in FDI firms, many of whom are internal migrants, this is the first experience of formal employment [16, 17]. These new employment possibilities for women could provide them with increased economic and career opportunities. However, women living in Ethiopia also must face high gender inequality regarding health and wellbeing [13]. Next to this already vulnerable position, the low wages and new roles as first-time formal employees (often in a new context due to internal migration) has further increases the vulnerable positions of women working in FDI in Ethiopia. Women working in FDI with a low socio-economic position might be at greater risk for a lower health and wellbeing, compared with their socio-economic more advantaged counterparts [5, 6]. To be able to decrease the socio-economic health and wellbeing inequities among women working in FDI in Ethiopia, it is crucial to gain insight into the underlying mechanisms that explain why such inequities exists.

### Explaining inequities in health and wellbeing

One relatively new theoretical model that has been shown to successfully explain socio-economic health inequities is the Reserve Capacity Model (RCM) [18–21]. The RCM explains how a low socio-economic position (SEP) results in health disparities over time and explicates the mediating role of ‘reserve capacities’ in the SEP and health status gradient. The RCM posits that individuals with a low SEP experience more daily hassles and major stressors in their lives and have lower reserve capacities compared with individuals with a higher SEP, leading to increased negative cognitions and emotions affecting their health and wellbeing outcomes [18, 22]. Three different types of reserve capacities are proposed by the RCM that can help people to deal with adversities and buffer against the negative effect of a low socio-economic position: intrapersonal reserves (e.g., psychological capital), interpersonal reserves (e.g., emotional and instrumental (e.g., financial) social support), and tangible reserves (e.g., assets such as having access to a toilet or a mobile phone) [18–21].

PsyCap is defined by Luthans and Youssef-Morgan [23] as a ‘positive psychological state of development characterized by: 1) having confidence and skills to take on and put in the necessary effort to succeed at challenging tasks (efficacy); 2) making positive attributions about succeeding now and in the future (optimism); 3) persevering towards goals and, when necessary, redirecting paths to goals in order to succeed (hope); 4) when beset with problems and adversity, sustaining and bouncing back and even beyond to attain success (resilience)’. High levels of PsyCap are associated with increased health and wellbeing outcomes in high-income Western countries [21, 24, 25] and middle-income income countries in Africa [26]. Furthermore, people with a low SEP have been shown to have lower levels of PsyCap than their high SEP counterparts, which has (partly) been shown to explain their increased number of health conditions and lower self-rated health outcomes [21].

Another reserve capacity that might be important when trying to explain the negative effects of a low SEP on health and wellbeing outcomes is the interpersonal reserve of social support. Social support is comprised of social interactions in which a person receives information, emotional or instrumental support [27, 28]. Social support has been shown to improve health and wellbeing and even to decrease morbidity and mortality rates both directly and indirectly, by protecting against the adverse effects of stressors on health [28–30]. Social support has shown similar associations with health and wellbeing in low- and middle-income countries in the Global South [31, 32]. Moreover, people with a low SEP often report lower-quality social networks and levels of social support compared with people with a high SEP [33], which may partly explain their worse health and wellbeing outcomes.

Although the RCM [18–20] posits that tangible reserve capacities, such as tangible assets (e.g., owning a mobile phone, having access to a toilet), mediate the negative association between SEP and health and wellbeing outcomes, not much research has thus far investigated these specific associations. The results of a study using a representative Dutch national sample [21] showed that lower financial-self-reliance could explain the negative association between SEP and self-reported health outcomes. Furthermore, this study showed that the mediation effects of the intrapersonal reserve PsyCap was stronger than the mediation effect of tangible resources. Moreover, since reserve capacities mediate (explain) the negative association between SEP and health and wellbeing, they could also act as a buffer for this negative association when increased through intervention efforts [21, 34].

Given the different cultural and economic context that countries in the Global South present relative to countries that have already been studied (high-income Western countries), it remains unclear whether the mechanisms explaining socio-economic health and wellbeing inequities as proposed by the RCM [18–20] work in the same way in low-income countries in the Global South. Scholars have found that people in Western high-income countries broadly tend to place a higher value on individual self-expression than on obligations to the group [35]. In contrast, in the Global South, societies are often organized around collectivistic values emphasizing relationships, group obligation, and interpersonal harmony [35–37]. The emphasis on interconnectedness in such societies might point to interpersonal reserves playing a stronger mediating role in explaining socio-economic health inequities than do intrapersonal reserves. Previous research has for instance indicated that, in collectivistic countries, social approval has the same predictive power towards wellbeing as do individual emotions, whereas in Western high-income countries, social approval was shown to play a less important role than emotions [38]. Furthermore, the buffering effect of social capital (an interpersonal reserve) showed to be stronger in more unequal and collectivistic societies [39], and for people with a lower socio-economic position [40, 41], compared with more equal societies and people with a higher socio-economic position, respectively. This additionally indicates that interpersonal reserves might play a larger explanatory role in health and wellbeing inequities among women working in FDI in Ethiopia.

The economic situation of a society or individual may also have implications for the extent to which reserve capacities can explain the socio-economic inequities associated with health and wellbeing. Several studies have confirmed that fulfillment of the basic needs (autonomy, relatedness, competence) increases wellbeing, even more so than perceived economic wealth [42–44]. Nevertheless, although in the multi-dimensional view on wellbeing, it is emphasized that economic growth or wealth should not be seen as the key determinants of a high wellbeing [42, 45], this only appears to be true once people are beyond the poverty level [42–44]. People in poverty experience financial scarcity, which has been shown to impede executive functions and encourage the discounting of future payoffs [46]. Experiencing financial scarcity may therefore result in not being able to fully make use of intrapersonal reserves such as PsyCap, since it might hinder making positive attributions about succeeding in the future (optimism) or having the possibilities to redirect pathways if needed (resilience). This emphasizes the stronger relevance of financial reserve capacities in low-income countries, where financial scarcity is experienced more often [47]. Previous studies have indeed shown that satisfaction of wealth was a stronger predictor of life satisfaction in poorer nations compared with wealthier ones [48], indicating that the value of tangible reserve capacities may be more critical in low-income countries where poverty levels are high. Moreover, when basic needs are not, or insufficiently, met, tangible reserves may be more pressing for wellbeing than for instance intrapersonal reserves [42–44].

### The current study

This study aims to investigate whether the RCM used to explain health and wellbeing inequities in Western high-income countries can be applied to a low-income country in the Global South. More specifically, we investigate whether different reserve capacities (intra-, inter-personal, and tangible) mediate or explain the association between relative SEP and multi-dimensional wellbeing and mental health problems. We specifically focus on Ethiopian women working in low-waged labor in apparel and floriculture sectors, since to decrease health and wellbeing inequities in a country, it is specifically important to focus on the more vulnerable populations [7]. Greater insight into which specific reserve capacities explain the negative association between relative SEP and multidimensional wellbeing and mental health problems is crucial for improving preventative strategies that aim to increase the wellbeing and mental health of this vulnerable group. This is especially relevant for the reserve capacities that can work as a buffer for the negative effect of relative SEP on multi-dimensional wellbeing and mental health and that can be easily increased by intervention efforts such as intrapersonal reserve Psychological Capital [25, 49]. The main research question therefore is ‘To what extent do (intra-, interpersonal, or tangible) reserve capacities explain the association between relative socio-economic position and wellbeing and health of women working in Ethiopia’s FDI-funded sectors?’. In collectivistic societies, the interconnectedness between people is more highly valued than individualistic societies [35]. Furthermore, in countries showing greater socio-economic inequalities and where more people experience financial scarcity (i.e., Ethiopia), the buffering effect of interpersonal reserves such as social capital might be greater than in countries that are more equal [39] and the value of money could be more pressing [42–44]. Therefore, we expect that, in the context of Ethiopia, interpersonal reserves and tangible reserves could play a more important role than intrapersonal reserves, considering the associations between SEP (independent variable) and mental health problems and wellbeing (dependent variables). More specifically, we expect interpersonal and tangible reserves to show stronger associations with the outcome measures and stronger mediation effects, compared with intrapersonal reserves.

## Method

### Sampling

This study is part of the larger “Women, Wellbeing and Work in Ethiopia” (3WE.nl) project, which aims to understand the wellbeing of women in Ethiopia’s export-oriented floriculture and apparel sectors. From November 2021 until December 2022, data were collected in two different regions of Ethiopia (Oromia (90.5%), and Southern Nations, Nationalities, and People’s region (9.5%)). The Amhara and Tigray regions were excluded, since large parts of these regions were at the epicenter of the civil war in the country at the time of data collection and therefore unsafe to travel to.

Participants were recruited from foreign-owned firms and farms in floriculture and apparel sectors in Ethiopia. The top ten largest employers were selected from a complete list of foreign-owned firms/farms registered by the Ethiopian Investment Commission and the Ministry of Trade and Industry. A stratified sampling design, stratified on an approximately equal distribution within OECD-ownership (no/yes) of the firms/farms and the sector type (apparel or floriculture) was used. We included 10 flower farms (*n* = 632), 11 apparel firms (*n* = 732) from OECD countries, and 8 flower farms (*n* = 580) and 8 apparel firms (*n* = 571) from non-OECD countries. Most participants (93%) were accessed via the firms/farms. To avoid selection bias, participants accessed via firms/farms were randomly selected from a list of all employees of the participating firms/farms by the interviewers until the quota was reached. Snowball sampling (7%) was used in case not enough participants could be interviewed in a specific firm/farm at the time of data collection. A total sample of 2,515 women participated in the study; 1,303 women working in the apparel and 1,212 women working in floriculture sector. There were 38 missing values (1.5%) on the measures relative socio-economic position (*n* = 37) and educational level (*n* = 1), because the women either answered ‘I do not know’ (*n* = 37) or did not want to indicate this (*n* = 1). Cases with missing values on important model variables were excluded. Since less than 1.5% (*n* = 38) of the cases showed missing values, list-wise deletion neither poses major threats to statistical power nor to biasing the results [50], which is why missing cases were not imputed. After excluding missing values, the analytic sample consisted of 2,477 women.

### Procedure

Ethical approval to conduct this study was granted by the Ethical Review Committee of the Inner City Faculties of Maastricht University (FASOS: ERCIC_172_22_01_2020). First, the selected farms and firms were contacted by telephone to ask for permission to approach women workers at their firm or farm to collect data. Only one apparel firm refused to participate, due to experiencing a heavy work load at the time the survey was conducted. The next apparel firm in the list was selected and included as a replacement. The questionnaires were translated into common local languages (Amharic and Affan-Oromo) and pretested. Interviewers were trained to interview the women workers using the predefined questionnaire in the preferred language of the participants. Interviews took about 60 min and most managers gave permission to conduct the interview in working hours. Participation was voluntary and informed consent was given before the interview started. Women received a 100 birr voucher (approximately 2 Euros) for their participation as compensation. The only exception is one farm which hosted several other surveys per year. Managers in this farm did not allow the fieldworkers to give an incentive to research participants.

### Measures

#### Mental health problems

The 12-Item General Health Questionnaire (GHQ-12) [51] is the most extensively used screening instrument for (non-psychiatric) mental health problems, and has also been used in studies in the Global South [52]. In this study it was used to measure the degree to which participants suffer from mental health problems. Each item assesses the relative severity of experienced mental problems over the past 4 weeks using a 4-point likert scale with response options ranging from 1 (“better than usual”) to 4 (“much less than usual”). Reverse coding was used for positively formulated items, meaning that higher scores indicate greater experience of mental health problems. Examples of items are: “Have you recently been able to concentrate on whatever you are doing?”, “Have you recently been feeling unhappy and depressed?”, “Have you recently lost sleep over worry?”, and “Have you recently been able to enjoy your normal day-to-day activities?”. Internal reliability (Cronbach’s alpha = .795) proved to be high and mean scores were used in the analyses.

#### Multi-dimensional wellbeing

Multi-dimensional wellbeing was measured using the 15-item PERMA-Profiler, which measures five domains of wellbeing (positive emotions, engagement, relationships, meaning, accomplishment) and has previously been shown to be a valid and reliable measure for wellbeing [53]. ‘Positive emotions’ refer to hedonic feelings of happiness (e.g., feeling joyful, content, and cheerful); ‘engagement’ refers to a psychological state in which people feel absorbed and focused on what they are doing, also referred to as ‘flow’ [54]: ‘positive relationships’ include feeling socially integrated, cared about and supported by others, and satisfied with one’s social connections; believing that one’s life is valuable and feeling connected to something greater than oneself is referred to by ‘meaning’; ‘accomplishment’ involves making progress toward goals, feeling capable to do daily activities, and having a sense of achievement [55, 56]. Response options ranged from ‘never’ (coded as 1) to ‘always’ (coded as 5). Examples of items are: ‘How much of the time do you feel you are making progress towards accomplishing your goals?’ (accomplishment), or ‘In general, to what extent do you lead a purposeful and meaningful life?’ (meaning). The scale showed a high internal consistency in our sample (Cronbach’s alpha = 0.844) and mean scores (higher scores indicate higher wellbeing) were used in the analyses.

#### Relative Socio-Economic Position (SEP)

Women were asked to assess the item ‘Compared to other people in your neighborhood, what would you say that your financial situation at the moment is?’ on a 4-point-likert scale with response options ranging from 1 (‘worse’) to 5 (‘better’) to measure their socio-economic position relative to other people in their neighborhood. The additional response option ‘I don’t know’ was coded as missing. This scale was derived from the MacArthur Scale of Subjective Social Status, a self-anchoring scale used to measure subjective social status and that has shown sufficient construct as well as face validity [57]. We adapted the McArthur Scale into a more simple scale that is more suitable for the included target population.

#### Psychological capital (PsyCap; intrapersonal reserve)

PsyCap was measured by the Compound Psychological Capital Scale Revised (CPC-12R) [58]. The scale included twelve items within four subscales (hope, optimism, resilience, and efficacy), using a 5- point Likert-scale ranging from 1 = ‘completely disagree’ to 5 = ‘completely agree’ (example items: ‘I am confident that I could deal efficiently with unexpected events’ (efficacy) or ‘I tend to bounce back quickly after serious life difficulties’ (resilience). Mean scores of the overall PsyCap construct were used in the analyses, with higher levels of PsyCap being indicated by higher scores. The CPC-12 showed high internal reliability within this study (Cronbach’s alpha = .798), comparable to the reference value (Cronbach’s alpha = .82) from Lorenz et al. [59].

#### Size of emotional social support network (interpersonal reserve)

The size of the emotional social support network was measured by a scale derived from previously conducted research [60]. Participants were asked about the size of their support networks ‘How many [family members, other relatives, neighbors, friends, co-workers, or other people] can you rely on in times of great personal needs (e.g., during illness, death in the family, etc.)?’. Participants had to provide the exact number of people within each possible group. A count variable was created by adding up the number of people provided for each item. Thereafter, a natural log transformation was used to adjust for the non-normal distribution of the variable.

#### Size financial social support network (interpersonal reserve)

The size of the financial social support network was measured by a scale derived from previously conducted research [60] that asks participants for different possible members of their supports network ‘How many [family members, other relatives, neighbors, friends, co-workers, or other people] can you rely on in case you have financial problems?’. Participants had to provide the exact number of people within each possible group. A count variable was created adding up the number of people provided for each item. Thereafter, a natural log transformation was used to adjust for the non-normal distribution of the variable.

#### Tangible assets (tangible reserve)

Tangible assets were measured by ten individual dichotomous (no = 0, 1 = yes) question derived from a previous research [60]. The scale includes questions asking participants whether they a) own one or several plots of land (agricultural land, plot for building a house); b) own the house they live in now; c) live in a house with more than one room; d) own a radio/tape recorder; e) own a TV; f) own a mobile phone; g) own a satellite dish; h) have access to tap water; i) have access to a toilet; j) have access to electricity. A count variable was created for each participant counting all times they indicated yes to these tangible resources. The count variable was included in the analyses, ranged from 0–10 with higher scores indicating more tangible assets.

#### Covariates

Age and highest attained educational level were included as covariates. Having sufficient money, the ability to give financial support, and frequency of migration were also included as covariates, since previous research has shown their association with wellbeing [42–44, 61]. Furthermore, whether the firm was owned by an owner from an OECD or non-OECD country and the type of firm (apparel or floriculture) were included as a covariates in all analyses.

Age was measured in years and included as a continuous variable in the model. Participants were asked about their highest attained educational level, included as a continuous variable in the analyses and recoded into the following response options (1 = illiterate and no formal education, 2 = no formal education but can read and write, 3 = completed first cycle of primary school, 4 = completed secondary cycle of primary school, 5 = completed preparatory school, 6 = completed Technical and Vocational Education and Training, 7 = completed college degree, 8 = completed university degree). The frequency of migration was measured through an item asking participants ‘How many other places did you live before your current place of residence?’. The number of other places/towns were included as a continuous variable in the model. Income sufficiency was assessed by the question ‘I currently have enough money to live on from day-to-day’ (no = 0/yes = 1). Ability to give financial support was measured using one item asking participants if they can lend money to other people (0 = no, 1 = yes). OECD-ownership (0 = non-OECD owned, 1 = OECD-owned) and sector type of the firm or farm (0 = apparel, 1 = floriculture) were also included as covariates.

### Design and analyses

**Fig. 1:**
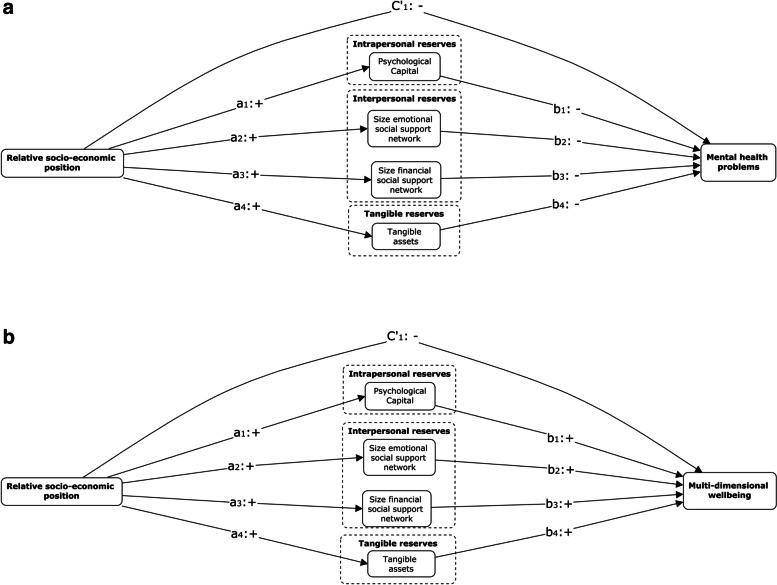
**a** Conceptual model mental health problems. *Footnote:* Covariates and co-variances between independent variables and mediators are not depicted in the visual representations but are included in the analyses; Analyses were performed adjusting for clustering (firms/farm). **b** Conceptual model multi-dimensional wellbeing. *Footnote:* Covariates and co-variances between independent variables and mediators are not depicted in the visual representations but are included in the analyses; Analyses were performed adjusting for clustering (firms/farm)

This study used a cross-sectional design. Descriptive analyses and Pearson correlations were calculated using IBM SPSS v28. Separate analyses were conducted for each outcome variable, to ensure that the number of free parameters did not exceed the included number of clusters (*n* = 37), thereby reducing the risk of model instability. To test the proposed conceptual models (see Fig. 1a and b for a visual representation), structural equation modelling was conducted using Mplus v7, adjusting for the clustered sampling design (firms/farms) in a two-level model and adjusting for age, frequency of migration, ability to give financial support, attained educational level, OECD-ownership and sector type of the firm or farm as covariates. Additionally, because bootstrap resampling cannot be combined with analyses adjusting for clustering, the same analyses testing the two conceptual models presented in Fig. 1a and b were repeated using bootstrap resampling with 1000 random draws (14) to test the robustness of the significance of the effects found in the cluster-adjusted structural equation mediation analyses.

## Results

### Sample description

The analytic sample consisted of 2,477 women with a mean age of 27.51 (SD = 7.93) years. Of all participating women, 11.1% indicated that they had no formal level of education (10.4% also indicated that they were illiterate), 12.0% had completed the first cycle (grades 1–4) and 26.8% the second cycle of primary school (grades 5–8), 29.3% indicated to have completed secondary school, 8.4% had completed preparatory school or Technical and Vocational Education and Training (TVET), and 12.2% had attained a college or University degree. The majority of women (82.2%) had migrated at least once in their lives. Of all women included in our study, 38.9% indicated that they had sufficient income to live on day-by-day and 28.7% indicated they had the ability to provide financial support to others (e.g., family members).

As data were collected from one source type (female workers) and using one method (survey), a post-hoc Harman’s single-factor test was conducted to test for the likelihood of common method variance bias [62]. Results showed only a 14.057% cumulative percentage of the sums of squared loadings on the first component (> 50% as a cut-off point indicating common method variance), indicating a very low likelihood of the presence of common method variance.

### Descriptive results

For an overview of mean scores, standard deviations and correlations between model variables, please see Table 1. Associations between model variables were all significant and in the expected directions (see Fig. 1a and b), except for the association between size of the emotional social support network and mental health problems. Furthermore, although a high positive correlation was found between size of the financial and emotional social support system, indicating that there is considerable overlap between the sizes of these two support systems, multi-collinearity diagnosis showed no reason for concern.

**Table 1:** Descriptive statistics of and Pearson and Point-biserial correlations between model variables

**Variable names**	**Mean (sd)**	**1.**	**2.**	**3.**	**4.**	**5.**	**6.**	**7.**	**8.**	**9.**	**10.**	**11.**	**12.**	**13.**	**14.**
**1. GHQ-12**^**a**^	1.99 (.41)	1													
**2. Wellbeing**	3.45 (.51)	-.273**	1												
**3. Income sufficiency**^**b**^** (0 = no, 1 = yes)**	-	-.231**	.261**	1											
**4. Relative SEP**	2.50 (.87)	-.224**	.283**	.328**	1										
**5. PsyCap**	3.90 (.40)	-.224**	.390**	.171**	.154**	1									
**6. Personal psychological support network**	11.20 (12.96)	.025	.120**	.125**	.183**	-.018	1								
**7. Financial social support network**	5.34 (5.47)	-.114**	.259**	.216**	.279**	.124**	.650**	1							
**8. Tangible assets**	5.26 (1.74)	-.074**	.168**	.252**	.191**	.085**	.130**	.144**	1						
**9. Age**	27.51 (7.93)	.046*	.013	-.020	-.041*	-.057**	-.010	-.083**	-.071**	1					
**10. Freq. Migration**	.99 (.65)	.123**	-.064**	-.031	-.119**	-.022	-.033	-.012	.027	-.011	1				
**11. Ability giving financial support**^**b**^** (0 = no, 1 = yes)**	-	-.124**	.141**	.226**	.179**	.050*	.260**	.272**	.073**	-.065**	.043*	1			
**12. Educational level**	3.56 (1.96)	-.047*	.033	.044*	-.012	.073**	.045*	.079**	.043*	-.375**	.036	.049*	1		
**13. OECD-ownership**^**b**^** (0 = no, 1 = yes)**	-	-.033	.002	.030	.032	-.012	-.049*	-.035	.061**	.055**	-.024	-.042*	.030	1	
**14. Sector type**^**a,b**^** (0 = apparel, 1 = floriculture)**	-	.094	.106**	.114**	.248**	.031	.124**	.171**	-.091**	.194**	-.038	.132**	-.421**	-.040*	1

^**^P < .01 (2-tailed)

^*^P < .05 (2-tailed)

^a^Higher scores indicate worse mental health

^b^For dichotomous variables point-biserial correlations are reported

### Results of cluster-adjusted structural equation modeling

#### Mental health problems

**Fig. 2:**
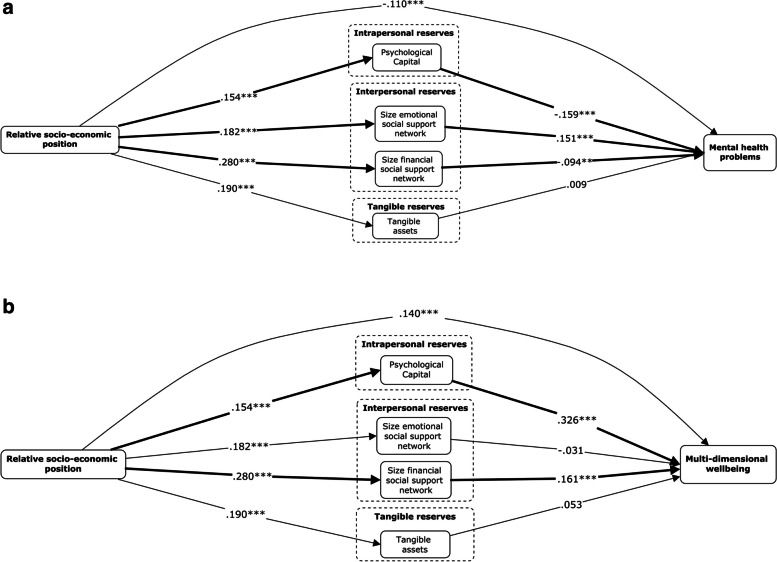
**a** Visualized results for mental health problems. *Footnote:* Covariates and co-variances between independent variables and mediators are not depicted in the visual representations but are included in the analyses; Analyses were performed adjusting for clustering (firms/farm); Bold lines indicate significant mediation effects; *** *p* < .001; ** *p* < .01; * *p* < .05. **b** Visualized results for multi-dimensional wellbeing. *Footnote:* Covariates and co-variances between independent variables and mediators are not depicted in the visual representations but are included in the analyses; Analyses were performed adjusting for clustering (firms/farm); Bold lines indicate significant mediation effects; *** *p* < .001; ** *p* < .01; * *p* < .05

The overall tested mediation model that used mental health problems as an outcome measure, explained 13.7% of the variance in mental health problems (see Table 2 in Additional materials and Fig. 2a) and shows a good fit when considering the standardized root mean square error residual (SRMR = .053) in combination with the root mean square error of approximation (RMSEA = .050) [63]. Although the comparative fit index (CFI = .821) does not exceed the threshold of .95, a reasonably acceptable threshold of .80 was reached [64].

The total effect of relative SEP on mental health problems showed a negative association that was significant (Std β = -.132, SE = .013, 95% CI -.199; -.065). The direct effect of relative SEP (Std β = -.110, SE = .026, 95% CI -.161; -.059) on mental health problems was also significant, even after controlling for the mediators in the model. Psychological capital (Std β = -.159, SE = .041, 95% CI -.239; -.079), and the size of the financial social support network (Std β = -.094, SE = .032, 95% CI -.157; -.031), showed a unique negative association with mental health problems; the higher the PsyCap and larger the financial social support network, the lower the degree of mental health problems. In contrast, the size of the emotional social support network (Std β = .151, SE = .024, 95% CI .103; .199) showed a significant, but unexpected, positive association with mental health problems, indicating that the larger the emotional social support network, the higher the mental health problems. Tangible assets (Std β = .009, SE = .035, 95% CI -.059; .077) did not show a significant association with mental health problems.

All four tested associations (see a_1_-a_4_ in Fig. 1a) between relative socio-economic position and reserve capacities, showed to be positive and statistically significant, with standardized β’s ranging from .154 (SE = .035; 95% CI .085; .224) for PsyCap to .280 (SE = .035; 95% CI .221; .337) for size of financial social support network.

Clustered adjusted model results showed significant mediation effects for psychological capital (Std β = -.025, SE = .011, 95% CI -.046; -.003) and for size of the financial social support network (Std β = -.026, SE = .010, 95% CI -.045; -.008) in the association between relative SEP and mental health problems. Also, the size of the emotional social support network showed a significant mediation result (Std β = .028, SE = .006, 95% CI .016; .039), however, not in the expected direction. Furthermore, the mediation result of the size of the emotional social support network also showed to be significantly different from the indirect effects via PsyCap or the size of the financial social support network (95% CI not overlapping). Tangible assets did not show to mediate the associations between relative socio-economic position and mental health problems. The additionally performed bootstrap analyses showed the same results and therefore show the robustness of mediation pathways found in the cluster-adjusted analyses (results can be obtained from the first author).

#### Multi-dimensional wellbeing

The overall tested cluster-adjusted mediation model using multidimensional wellbeing as an outcome measure explained 23.5% of the variance in wellbeing (see Table 2 in Additional materials and Fig. 2b) and shows a good fit when looking at the standardized root mean square residual (SRMR = .05) combined with the root mean square error of approximation(RMSEA = .050) [63]. Even though the comparative fit index (CFI = .840) does not exceed the .95 (threshold value), it still meets the requirements of a reasonably acceptable threshold of .80 [64].

The total effect of relative SEP on multi-dimensional wellbeing showed a positive association that was significant (Std β = .239, SE = .021, 95% CI .151; 328). The direct effect of relative socio-economic position (Std β = .140, SE = .035, 95% CI .071; .208) on multi-dimensional wellbeing stayed significant, even after additionally controlling for the mediators. Psychological capital (Std β = .326, SE = .035, 95% CI .256; .396) and the size of the financial social support network (Std β = .161, SE = .032, 95% CI .097; .224) also showed a positive association with multi-dimensional wellbeing. Tangible assets (Std β = .053, SE = .028, 95% CI -.002; .109) and size of emotional social support network (Std β = -.031, SE = .037, 95% CI -.104; .042) did not show a significant association with multi-dimensional wellbeing.

Clustered adjusted model results showed significant mediation effects similar in strength (95% CI overlapping) for psychological capital (Std β = .050, SE = .015, 95% CI .020; .080) and size of the financial social support network (Std β = .045, SE = .012, 95% CI .021; .068) in the association between relative SEP and multi-dimensional wellbeing. Tangible assets and the size of the emotional social support network did not show to mediate the association between relative socio-economic position and multidimensional wellbeing. The additionally tested model that included bootstrap analyses showed the same results as the cluster-adjusted model, and therefore the robustness of mediation pathways found in the cluster-adjusted analyses (results can be obtained from the first author).

## Discussion

The main aim of this study was to investigate whether the Reserve Capacity Model (RCM) that explains health and wellbeing inequities, can be applied in the context of Ethiopia, a low-income country in the Global South. We expected different reserve capacities (i.e., interpersonal and tangible, in contrast to intrapersonal) to play a more important role in explaining mental health problems and wellbeing inequities in the Ethiopian context than in the Western context, due to the higher value given to social interconnectedness [35]. Three out of four (for mental health problems) and two out of four (for multi-dimensional wellbeing) tested indirect pathways were significant. This indicates that the RCM can be applied to the Ethiopian context, but not perfectly. First, contrary to what was expected, instead of interpersonal or tangible reserves (path b2-b4 in Fig. 1a and b), the intrapersonal reserve PsyCap showed the greatest association (path b1 in Fig. 1a and b) with mental health problems and multi-dimensional wellbeing. This is in line with results obtained in previously conducted studies in Western high-income countries (individualistic societies) [21, 34]. Thus, also in the Ethiopian context, higher PsyCap scores (intrapersonal reserve capacity) were more strongly associated with lower reports of mental health problems (protective) and higher multi-dimensional wellbeing scores as compared to interpersonal or tangible reserve capacities. This may indicate different things. Perhaps in the Ethiopian context the society’s general prioritization of individual needs have grown, due to steep economic growth, the influx of foreign capital, and the arrival of neo-liberal economic values. Indeed, there is empirical evidence that individualism has increased globally due to socio-economic development [65]. Another explanation could be that, although PsyCap was measured on the individual level and in the exact same way as in studies conducted in high-income countries, the items of this questionnaire measuring hope, efficacy, resilience and optimism were interpreted by participants in ways that cannot be seen independently from their group values. Nevertheless, it is important to note that, although PsyCap (intrapersonal reserve) showed the strongest association with wellbeing and mental health problems, the indirect effects found for Psycap and size of the financial support network (interpersonal reserve) did not differ significantly. Thus, in Ethiopia, intrapersonal reserves do not seem to explain the socio-economic difference found in wellbeing and mental health outcomes, to a greater extent than interpersonal reserves. The second unexpected result was the insignificant association between tangible assets and mental health problems and wellbeing scores. This also contradicts the findings of studies conducted in high-income counties, where tangible reserves have been shown to mediate the association between SEP and health outcomes [21]. Although no significant results for the number of tangible assets was found, future research should further investigate the importance of tangible reserves not assessed in the current study. For example, the importance of having access to safe transportation between work and home for experienced wellbeing has been suggested by previous studies [66]. Also, the possible downstream consequences of having access to tangible reserves should be considered in future research. Tangible reserves may not be ubiquitously positive, but could possibly lead to jealousy, the need for protection from theft, and personal security risks, especially if these tangible reserves are scarce. Third, the size of the emotional social support network (interpersonal reserve capacity) showed an unexpected positive association with mental health problems, indicating that the size of the emotional social support network could even have detrimental effects on mental health in the Global South. This result contrasts with findings from high-income countries, where both, emotional and financial social support has been linked not only to increased mental health but also to both decreased morbidity and mortality [30]. One explanation for these unexpected results could be the low-income country-context with strong emphasis on group values, where being able to support others in times of need (psychologically, but even more importantly financially) has been shown to be important for women’s mental health, especially when it involves family members [45, 67, 68]. The results of the present study indeed indicate that being able to support others financially directly reduces mental health suffering. However, when the size of emotional social support network increases, women might feel a stronger social expectation to provide also financially to a larger group of people. Such social expectations (social norms) could increase negative feelings (e.g., guilt, shame, or an increased burden of responsibility). Since most of the women are not able to provide financially for others (in the current study, the figure was 28.7%), this low ability may be perceived as a burden and may increase feelings of being socially disapproved of. Previous research has indeed shown that norms related to social approval are more strongly associated with wellbeing outcomes in countries in the Global South than in Western countries [38].

### Strengths, limitations and suggestions for future research

This study is the first quantitative study to test the RCM in the context of Ethiopia (a low-income country in the Global South) using a large sample size and including different FDI sectors, indicating that results can be generalized to Ethiopian women working in the foreign-owned apparel and floriculture sectors. Nevertheless, this study also has limitations. First, although the associations and mediation pathways tested in the current study are based on theory [19, 20, 22], cross-sectional data were used and therefore no causal inferences between model variables can be made. Reversed causality between reserve capacities and mental health, and between wellbeing outcomes and relative SEP could be possible. For instance, a large financial social support network could lead to a higher perceived socio-economic position, or, lower mental health could lead to fewer financial means due to illness and in turn a lower relative SEP. Although this study is the first to provide insight into which reserve capacities are specifically important in Ethiopia, future longitudinal studies are needed to further investigate the directions of the tested associations.

Secondly, neither the composition of the people in the emotional social support network, nor the quality of the relationships was measured in the current study. It might be that participants feel a stronger obligation to provide financially to (direct) family members than to non-family members of their emotional social support network and that an inability to do so leads to stronger feelings of guilt and maybe even shame, emotions that have shown to negatively affect mental health [69]. Furthermore, participants who have greater emotional social support networks might have lower quality relationships within their network, which could explain why the size of the emotional social support network has a detrimental effect on mental health. Future studies should therefore also consider the composition of people in respondents’ emotional social support networks, as well as the quality of these relationships.

Third, although previous studies have shown similar explained variances when testing similar models [21], only 13.7% of the variance in mental health problems, and almost 24% of the variance in multi-dimensional wellbeing, is explained by the models tested in the current study. This indicates that we may have missed other important determinants of mental health problems and multi-dimensional wellbeing. Future research should determine which other determinants (e.g., political instability, perceived neighborhood disorder) are important.

Last, the direct effect of relative SEP remained significant after all tested mediators and covariates were included. This indicates that the association between relative SEP and mental health problems or multi-dimensional wellbeing is not fully explained by the included mediators. Future research should look into other potential reserve capacities (such as access to transportation [66]) that may be able to explain the remaining effect of socio-economic position on mental health problems and multi-dimensional wellbeing.

### Practical implications

Gaining more insights into the applicability of theoretical models explaining health and wellbeing inequities in this specific context, could also provide useful input for effective preventative efforts aiming to reduce these inequities among women living and working in the Global South. The results of this study indicate that intervention efforts that focus on increasing PsyCap, increasing financial means and strengthening financial support networks could serve as a buffer against the negative associations between a low perceived SEP and higher mental health problems and lower wellbeing outcome measures among women working in FDI. PsyCap has proven to be malleable in micro-interventions [23, 59, 70] and has shown promising results in increasing performance and life satisfaction [71]; it could therefore be a promising avenue for prevention. The size of the financial support network is another reserve capacity that shows a protective effect for a low SEP, which may be increased by facilitating personal loans that could support women through challenging financial times. Furthermore, since being able to provide financially for others and having sufficient income directly impact wellbeing and mental health in a positive way, intervention efforts should additionally be directed towards creating decent pay (a living wage) for these women working in apparel and floriculture sectors in the Global South.

## Conclusion

This study provides evidence for the theoretical relevance of the Reserve Capacity Model that reaches beyond Western countries to the Ethiopian context, a low-income country in the Global South. In Ethiopia, PsyCap (consisting of hope, efficacy, resilience, and optimism) and size of the financial social support network seem to be important reserve capacities that could protect against socio-economic mental health inequities among women working in FDI firms. On the other hand, tangible resources (tangible assets) did not show to explain socio-economic inequities in both outcome measures (multi-dimensional wellbeing and mental health problems). The size of the emotional social support network was able to explain socio-economic inequities in mental health problems only, and actually appeared to have a detrimental effect on mental health. To decrease mental health and wellbeing socio-economic inequities among women working in Ethiopia’s apparel and floriculture sectors, prevention efforts should therefore try to increase PsyCap, size of the financial support networks and wages, especially for women with a low SEP.

### Supplementary Information

**Supplementary Material 1.**

## Data Availability

The data used are available from the PI of the 3WE project (Prof. V. Mazzucato: v.mazzucato@maastrichtuniversity.nl) on reasonable request and after the project has been finalized (Nov. 2023).

## Abbreviations

SDG: Sustainable Development Goal
FDI: Foreign Direct Investment
SEP: Socio-economic position
PsyCap: Psychological capital
GHQ: General Health Questionnaire
