# Supplementary material for: Socio-economic inequities in mental health problems and wellbeing among women working in the apparel and floriculture sectors: testing the mediating role of psychological capital, social support and tangible assets
Source: BMC Public Health. 2024 Apr 25;24:1157. doi: 10.1186/s12889-024-18678-5 (PMC11044536; doi:10.1186/s12889-024-18678-5)
Supplement: Supplementary file 1 — Supplementary Material 1. [file 12889_2024_18678_MOESM1_ESM.docx]

**Table 2** Standardized results of the cluster-adjusted mediation analyses (N=2,477)

| **Dependent variables** | **GHQ-12 mental health** | | **Multi-dimensional wellbeing** | | |
| --- | --- | --- | --- | --- | --- |
|  | **β (SE)** | | **β (SE)** | | |
| Relative SEP (C’_1_) | -.110*** (.026) | | .140*** (.035) | | |
| PsyCap (b_1_) | -.159*** (.041) | | .326*** (.035) | | |
| Size emotional social support network (b_2_) | .151*** (.024) | | -.031 (.037) | | |
| Size financial social supportive network (b_3_) | -.094** (.032) | | .161*** (.033) | | |
| Tangible assets (b_4_) | .009 (.035) | | .053 (.028) | | |
| Age | .016 (.024) | | .047* (.024) | | |
| Frequency of migration | .099*** (.019) | | -.028 (.016) | | |
| Attained educational level | -.065* (.026) | | .019 (.019) | | |
| Income sufficiency (no=0, 1=yes) | -.135*** (.037) | | .109*** (.026) | | |
| Ability giving financial social support (no=0, 1=yes) | -.071*** (.020) | | .028 (.024) | | |
| OECD-ownership (no=0, 1=yes) | -.027 (.057) | | -.003 (.052) | | |
| Sector type (0=apparel, 1=floriculture) | .093 (.056) | | -.020 (.048) | | |
| **Mediators (reserve capacities)** | **PsyCap (a1)** | **Size emotional social support network (a2)** | **Size financial social support network (a3)** | **Tangible assets**  **(a4)** |  |
|  | **β (SE)** | **β (SE)** | **β (SE)** | **β (SE)** |  |
| Relative SEP | .154*** (.035) | .182***  (.037) | .280***  (.035) | .190*** (.028) |  |
| **Indirect associations relative SEP on DVs via reserve capacities (a_1-4_-*b_1-4_)** | **GHQ-12 mental health** | | **Multi-dimensional wellbeing** | | |
|  | β (SE) | | β (SE) | | |
| PsyCap | -.025* (.011) | | .050** (.015) | | |
| Size emotional social support network | .028*** (.006) | | -.006 (.007) | | |
| Size financial social support network | -.026** (.010) | | .045*** (.012) | | |
| Tangible assets | .002 (.007) | | .010 (.006) | | |
| *Footnote:* *** p < .001; ** p < .01; * p < .05; SE = standard error; a1-a4, b1-4, and C’corresponds with the different pathways tested in the two analytic models presented in Figure 1a and 1b. | | | | | |
